# Data on a new neurorehabilitation approach targeting functional recovery in stroke patients

**DOI:** 10.1016/j.dib.2019.104685

**Published:** 2019-10-28

**Authors:** Loris Pignolo, Sebastiano Serra, Giuseppina Basta, Simone Carozzo, Francesco Arcuri, Luigina Maria Pignataro, Irene Ciancarelli, Paolo Tonin, Antonio Cerasa

**Affiliations:** a S. Anna Institute, Research in Advanced Neurorehabilitation (RAN), 88900, Crotone, Italy; b Department of Life, Health and Environmental Sciences, University of L'Aquila, Nova Salus S.r.l., L'Aquila, Italy; c IBFM, National Research Council, 88100, Catanzaro, Italy

**Keywords:** Stroke, Robotic neurorehabilitation, Integrated system, Upper and lower limbs

## Abstract

Robotic-assisted devices are known to positively affect the recovery of one specific motor effector after stroke. However, it has widely been reported that the functional status of patients is only partially ameliorated after application of this kind of advanced treatment.

Here, data about the effect of a new rehabilitation approach has been described in a large population of stroke patients. We sought to validate an integrated rehabilitation system for stroke (IRSS) patients, which is composed of a set of robotic-assisted tools aimed at recovering the entire body.

We evaluated the motor recovery in 84 stroke patients equally divided into experimental and control groups to assess the difference between IRSS approach and conventional rehabilitation treatment. We found that IRSS induced a significant improvement as measured by functional neurological scales, such as the barthel index and functional independence measure.

The data provided in this article will assist therapists and physicians working for developing new rehabilitation protocols more focused on a holistic functional recovery approach. The data are available at Mendeley Data, https://doi.org/10.17632/wptmgm7zk2.1.

**Table:** Specifications Table

Subject area	Biomedical Engineering
Specific subject area	Robot-assisted neurorehabilitation for recovery of motor functions in stroke patients
Type of data	Table, Excel file
How data was acquired	Data were acquired using standardized clinical measures including: the Barthel Index; Motricity Index (MI); Fugl-Meyer scale test (FM-UE); Functional Independence Measure (FIM) and the Trunk Control Test (TCT). See Supplementary Material
Data format	Analysed
Parameters for data collection	Samples were carefully enrolled following several clinical criteria. Scores extracted from well-known and internationally validated clinical scales were employed. SPSS (Statistical Packages for Social Science Students) was used in determining pattern of changes in clinical evaluations before and after the proposed neurorehabilitation protocol
Description of data collection	Clinical assessments were performed by blinded physicians before and after an 8-weeks neurorehabilitation treatment.
Data source location	S. Anna Institute and Research in Advanced Neurorehabilitation; Crotone, Italy
Data accessibility	With the article

**Table:** 

**Value of Data** • The data recorded after motor treatment performed with our integrated robotic rehabilitation system reveal a larger functional recovery than the conventional approach. • The data could serve as a benchmark for other researches to assess the improvement in functionalities of stroke patients • The data could be used in the development of further experiments on the validation of other robotic neurorehabilitation systems.

## 1 Data

**Fig. 1:**
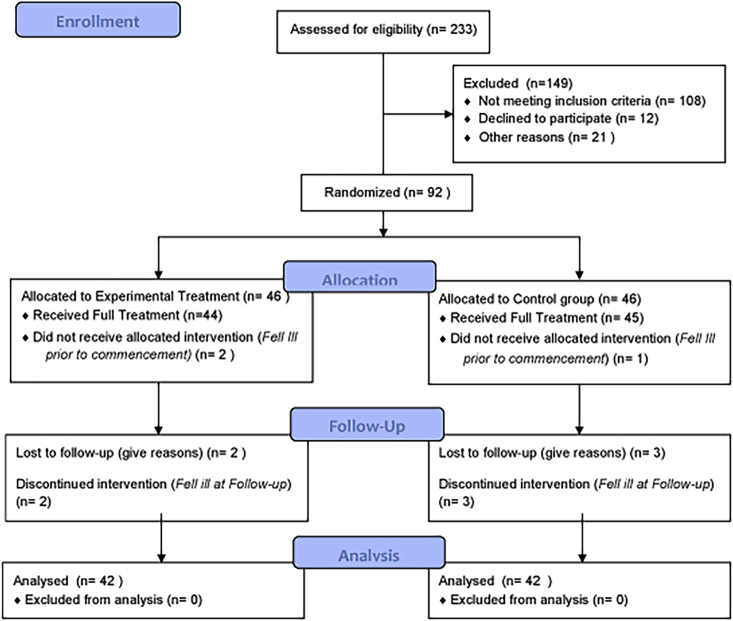
CONSORT Flow diagram showing the phases of a parallel randomised trial of two groups of patients with stroke underwent experimental or conventional motor rehabilitation treatments.

These data (available at doi:10.17632/wptmgm7zk2.1) describe the effectiveness of a series of tools for neurorehabilitation, defined as Integrated Robotic System for Stroke (IRSS). In particular, the proposed model was based on a holistic approach to motor disorders aimed at recovering the functional status of stroke patients. Using well-known clinical scales for assessing motor recovery (see Supplementary Materials) we evaluated the effects of the IRSS on a large sample of patients with stroke divided into experimental and control group (see Fig. 1).

From an initial cohort of 233 subacute hemiplegic patients, after severe inclusion criteria data, 84 patients were included in the final analysis phase. At baseline, the vast majority of patients had a moderate range of impairment similar between the two groups (all *p*'s > 0.4). Moreover, no significant differences in terms of age, sex, the time elapsed from episode and length of stay in the intensive rehabilitation unit were detected between groups (see Table 1). The hemiplegic side was properly disturbed among patients (50% left side).

**Table 1:** Participant's characteristics.

Variables	Experimental Group	Control Group	*p* values
Gender (m/f)	31/11	31/11	
Age (years)	69 ± 10.5	69.3 ± 10.6	*.67*
Time from Lesion (days)	14.1 ± 9.4	15.4 ± 5.9	*.96*
Days in Rehabilitation Unit	61.1 ± 19.9	54.5 ± 18.2	*.77*

Data are given as mean values (SD) or median values (range) when appropriate.

**Fig. 2:**
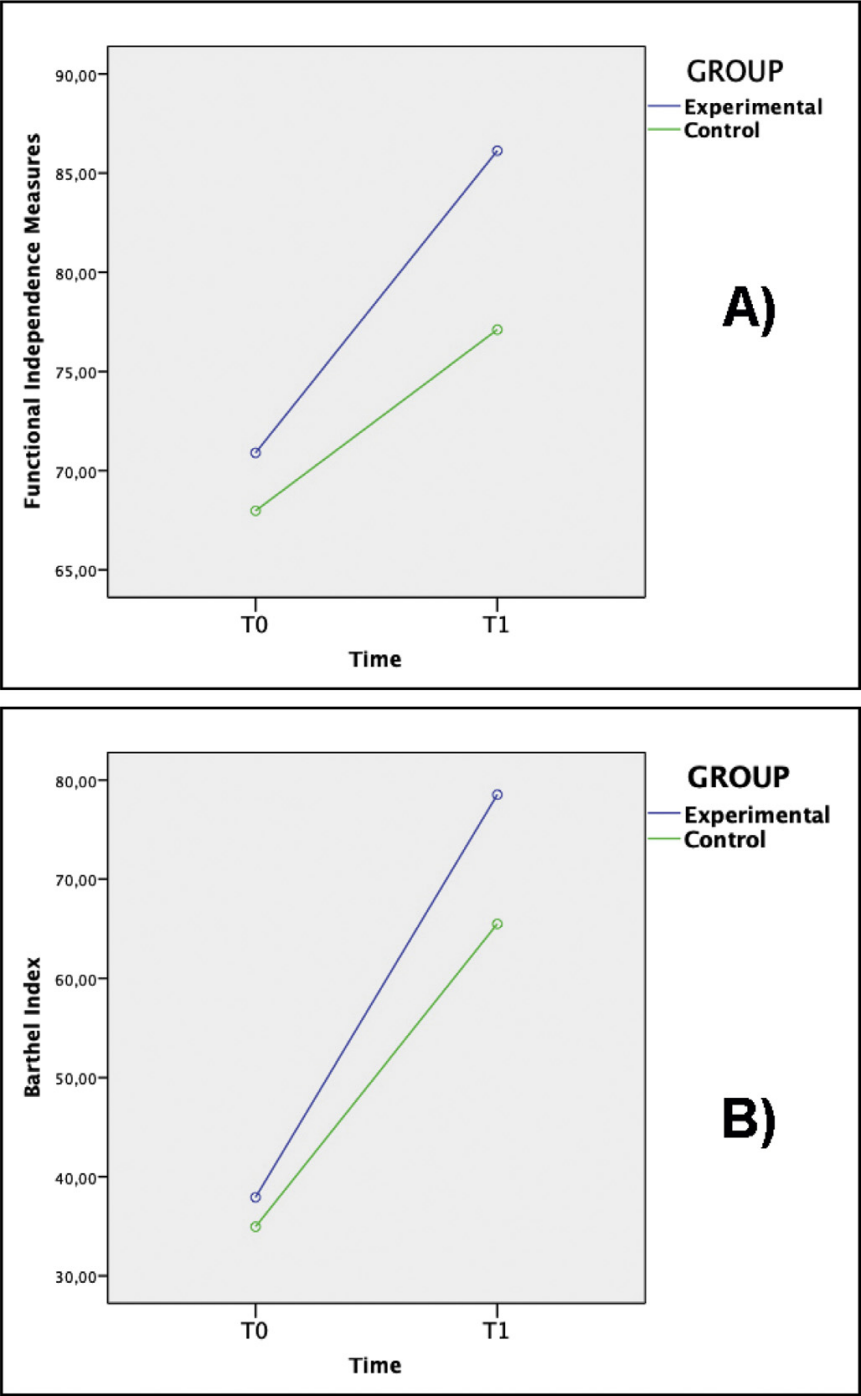
Motor effects of the integrated rehabilitation system for stroke (IRSS). ANOVA analysis revealed a significant improvement in the experimental group with respect to the demographically-/clinically-matched control group during the re-test phase (T1) versus the baseline evaluation (T0 phase). We detected functional recovery in the: A) Functional Independence Measures (FIM) and B) Barthel Index (BI).

As shown in Table 2, both treatments induced evident motor improvement in stroke patients, independently from the group. A group × time interaction effect was only detected in the FIM and BI scales (Fig. 2), where the experimental group showed a significantly greater improvement with respect to control group (Fig. 2) (*F*_*1,78*_-value = 8.3; p < 0.005; *F*_*1,78*_-value = 9.1; p < 0.003; respectively). Effect size analysis confirmed the presence of moderate effects for these factors.

**Table 2:** Measures of motor recovery in the experimental and control groups after rehabilitation.

Clinical measures	Baseline (T0)		Re-test (T1)		Effect Size^a^	Statistics^a^
	Experimental Group	Control Group	Experimental Group	Control Group	*Cohen's d*	*p-values*
FM-UE	44.6 ± 20.1	53.5 ± 26.1	74.4 ± 24.4	76.4 ± 28.9	.35	*.13*
FIM	57.7 ± 22.8	51.8 ± 23.9	96.6 ± 21	78.5 ± 23.9	.65	*.005*
BI	27.9 ± 23.4	23.6 ± 23	74.5 ± 25.2	56.4 ± 26.7	.66	*.003*
TCT	38.1 ± 24.7	34.9 ± 26	78.5 ± 25.3	65.4 ± 25.2	.48	*.11*
MI	71.6 ± 28.7	67.8 ± 36.2	86.1 ± 18.3	77.1 ± 32.4	.38	*.07*

FM-UE: Upper Extremity Fugl-Meyer scale test. FIM: Functional Independence Measures.

BI: Barthel Index.

TCT: Trunk Control Test.

MI: Motricity Index.

a Effect Size and *p*-values refer to between group comparisons.

**Fig. 3:**
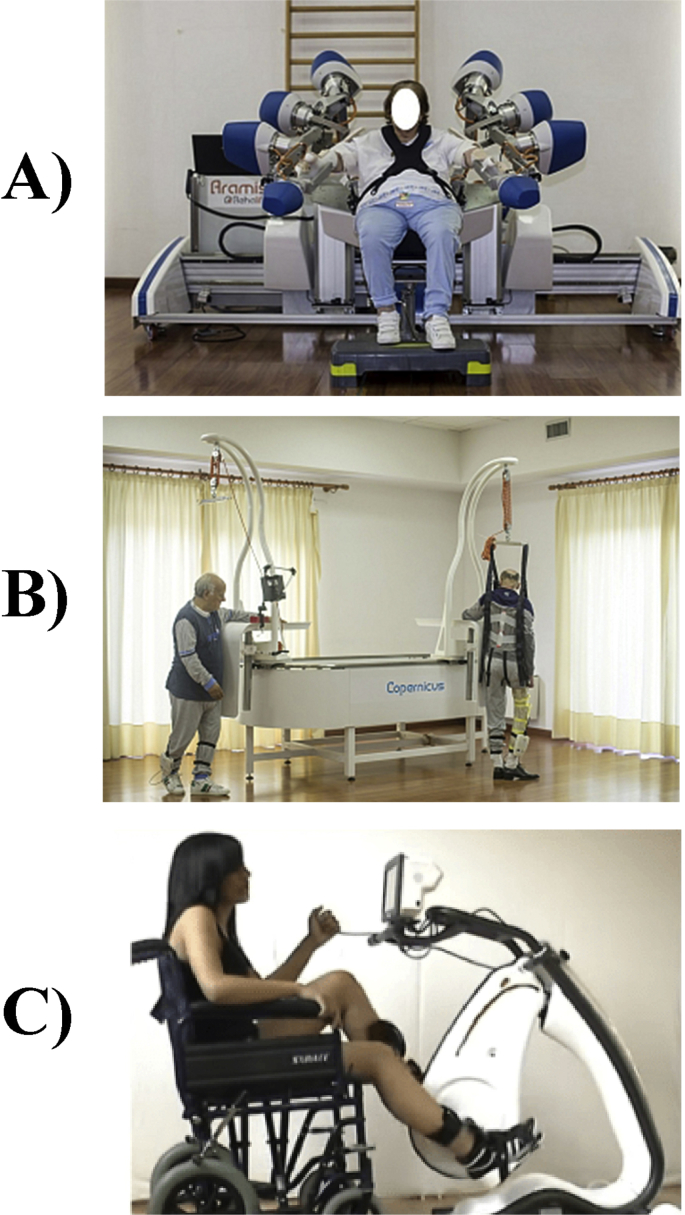
The three robotic devices employed for the integrated rehabilitation system for stroke patients (IRSS) covering the recovery of the entire body: A) ARAMIS; B) Copernicus; C) Pegaso.

In summary, we can affirm that IRSS is a new promising approach to induce significant motor improvements in stroke patients undergoing end-effector robotic rehabilitation (Fig. 3). Therefore, these data could be used in the development of new approaches more focused at recovering the functional status of stroke patients besides the distal segment of an impaired limb.

## 2 Experimental design, materials and methods

### 2.1 Participants

The first participant was randomized in January 2009 and the last on December 2017. There were two dropouts in the experimental group (two before beginning the program) and two participants were lost to follow up. A similar number of dropouts occurred in the control group (three before beginning the program). We enrolled all patients who met the criteria for the first attack of sub-cortical ischemic stroke recruited at S. Anna rehabilitation Center. From an initial cohort of 233 subacute hemiplegics, we enrolled only those patients who fulfilled the following criteria: (i) unilateral stroke, (ii) ability to follow verbal instructions; (iii) eight-handed patients. Exclusion criteria were (1) bilateral impairment; severe sensory deficits in the paretic upper limb; (2) pregnancy, epilepsy, aphasia, cognitive impairment (Mini Mental State Evaluation, MMSE < 24) or behavioural dysfunction that would influence the patient's ability to comprehend or participate in the treatment; (3) botulinum toxin injections or other medication influencing the function of the upper-limb; (4) inability to provide informed consent and (5) and/or pacemakers or other metallic implants incompatible with the 3T MRI scanner. From the initial cohort, 92 patients were selected for the rehabilitation program.

All the participants gave written informed consent. The study was approved by the ethical committee of the University ‘‘Magna Graecia’’ of Catanzaro, according to the Helsinki declaration.

### 2.2 Assessment

Baseline and post-intervention assessments were each completed in two phases called T0 & T1. The primary outcome was the clinical performance recorded with an extensive series of standardized tests (see Supplementary Material) administered by an experienced neurologist with more than 20 years' experience in clinical rehabilitation, blind to any other result. The degree of disability during activities of daily living was assessed with the Barthel Index (BI [1]) and motor strength of the upper-limb with the Motricity Index (MI) [2]. Patients’ synergistic motor control of the paretic arm was assessed with the upper extremity (UE) section of the Fugl-Meyer scale test (FM-UE) [3]. Further measures included the Functional Independence Measure (FIM) [4] and the Trunk Control Test (TCT) [5]. These assessments were completed in random counterbalanced order and took approximately 1 h to complete. Baseline and follow-up assessments were conducted within 1–2 days prior to training commencement and after intervention finishing.

### *2.3* *Procedure*

Following previously validated protocol [6], we performed a double-blind randomized controlled trial divided into 5 principal stages. The first stage was based on the recruitment of the patients for the study (see inclusion criteria reported above). Physicians (carrying out the clinical baseline assessment [T0] and post-treatment investigation [T1]), as well as, the primary researcher and data entry assistants were all blinded to the group membership of the patients. In the second stage, the eligible stroke patients underwent clinical examination (T0). In the third stage, participants were randomly assigned to 2 groups (experimental or control) using a computer-generated, site-stratified, randomization schedule. Randomization was stratified according to age and sex. For each stratum, random numbers were assigned to the participants and put into envelopes; it was determined randomly whether the even or odd number would enter the experimental group. Participants were assigned to the study according to the numbers they received on opening the envelopes. The different steps in this process were administered by different research assistants who were blinded to the other processes. In the fourth stage, participants underwent rehabilitation programs consisting of both, conventional and synergic-assisted neurorehabilitation activities. All rehabilitation sessions were performed in the morning. Treatments were carried on by (blind) expert therapists. To facilitate blinding, participants were told that each session involved different computerized activities but were not explicitly told of a treatment or control group. The duration of treatment and the intensity of treatments were the same for each group. Finally, at the end of treatment, participants from both groups were given a blind evaluation, using the same protocol as at a baseline.

### 2.4 Interventions

Both experimental and control groups attended rehabilitation sessions at the S. Anna rehabilitation center and both conditions were matched in terms of daily time spent and intensity. Both approaches consisted of intensive 3-h sessions, five weekly over 2 months.

The experimental group underwent the IRSS protocol, which consisted of combined sessions of three different end-effector robotic devices (Fig. 3):

a) The Automatic Recovery Arm Motility Integrated System (**ARAMIS**) is a concept robot and prototype for the neurorehabilitation of the paretic upper limb developed at the Institute S. Anna—Crotone, Italy (http://www.rehalife.it/en/products). ARAMIS was designed with two computer-controlled, symmetric and interacting exoskeletons, which compensate for the inadequate strength and accuracy of the paretic arm movements and the effect of gravity during rehabilitation. The basic idea is to exploit proprioceptive inputs using passive, repetitive, interactive, high-intensive bilateral movement training, which has been demonstrated to enhance motor recovery in stroke patients [[7], [8], [9], [10]]. This device has been widely validated (Colizzi et al., 2009; Dolce et al., 2009; Pignolo et al., 2012) with respect to conventional neurorehabilitation approaches. In agreement with previously validated version [6,[11], [12], [13]], the ARAMIS protocol for rehabilitation included 60-min sessions over periods not exceeding 7 weeks.
b) **Copernicus** is a system for the correct load balancing aimed at obtaining an early start of locomotion (http://www.rehalife.it/en/products). The patient wears a pair of insoles in his or her shoes equipped with piezoresistive sensors that detect the support of the foot and transfer the information via Wi-Fi to a controller with a monitor (tablet) that is visual feedback for the patient when executing the various rehabilitation exercises. Under the constant assistance of the therapist, the patient is positioned in an upright position laterally to mechanical support that is adjustable in height to position the arm in 90° abduction with respect to the trunk and that also acts as lateral support to fix the healthy limb's hip. This way the patient not only is assisted by the therapist but also feels solid support for the healthy hemi-side. In a first rehabilitation phase, the patient wears a sling that supports him or her from above to relieve the weight and to guarantee the standing position. The use of the sling also avoids the risk of falls and guarantees that the exercises are done in maximum safety for the patient. In a second rehabilitation phase, the patient can start locomotion as the arm-hip support of the healthy hemi-side can slide along a mechanical track so the patient can make a circular path and be supported, if needed, by the sling as it is also fixed onto the sliding support. The rehabilitative exercises consist of a first mode in which the activity required to the patient is to distribute the weight by progressively alternating the load from one foot to the other. The sensor insoles measure the support times for each foot and the number of support changes. Besides, for each foot, it is possible to extrapolate if the foot support is uniform as each sole has three sensors in three specific positions to support the foot. A sensor is positioned in the foot's inner plant, one is for the foot's external plant and one is for the heel. This aspect can also be displayed on the tablet monitor so the patient can instantly correct the support. The second modality of rehabilitative exercises consists of walking and the patient can progressively reach a predetermined number of steps by displaying a virtual landscape path on the tablet monitor. The Copernicus protocol for rehabilitation included 60-min sessions over periods not exceeding 8 weeks.
c) **Pegaso** is a motorized cycle ergometer with functional electrical stimulation, for the rehabilitation of both the lower and upper limbs (http://www.biotechrehabilitation.com/en/pegaso-fes-cycling/pegaso-fes-cycling). Pegaso is equipped with an electronic control system capable of recognizing the level of muscular effort of the subject to have the most suited level of exercise for the physical state of the patient. In addition to allowing the pedalling movement and to adjust its resistance for the cardio-pulmonary exercise, Pegaso is able to keep under control and accurately measure the kinematic and dynamic parameters of the movement, like the pedalling speed, the power exerted by the muscles, the virtually travelled distance. The system is equipped with an electrostimulator with 6 independent channels, each of which can deliver up to 140 mA. Lastly, the PEGASO controller automatically regulates, at every moment of the therapeutic session, the intensity of electrical stimulation and therefore the work done by the muscles, and the help or resistance opposed by the motor to the pedalling to optimize the exercise in each pathology condition and state of training the Pegaso protocol for rehabilitation included 60-min sessions over periods not exceeding 8 weeks.

Otherwise, the control group underwent conventional treatment consisting of exercises for passive and active mobilization of upper and lower limbs, trunk control, standing, deambulation. All patients also participated in the program of occupational therapy to promote recovery of autonomy in everyday life. Time and intensity of conventional treatment were similar to IRSS protocol.

### 2.5 Statistical analysis

Statistical analyses were performed with SPSS (Statistical Package for Social Sciences, version 12.0, http://www.spss.it/). Demographic differences between groups were tested using the unpaired *t*-test. The analysis for changes in clinical variables between baseline and after treatment was performed using the General linear mixed model (GLM) for repeated measures to test whether these variables differed across time in the groups. Factors were: group (Experimental Vs Control) and time (T0 Vs T1) and we obtained effects from each of them together with the interaction between group and time for each of the variables. The magnitude of change resulting from the intervention was obtained using Cohens *d* based on delta scores calculated as the differential between T1 − T0 scores. Generally, an effect size value of 0.2 is considered a small effect, 0.5 is considered a medium effect, and values of 0.8 and above are considered large effects. All statistical analyses had 2-tailed α levels of < 0.05 for defining significance.

## Author contributions

All authors gave a substantial contribution to conception and experimental design, as well as data analysis, interpretation and drafting of the manuscript. The submission of this paper has been approved by all authors and by the institution where the work was carried out.

## Funding

This study was supported by MIUR, Italy.

## References

[1] Collin C., Wade D.T., Davies S., Horne V. (1988). The Barthel ADL Index: a reliability study. Int. Disabil. Stud..

[2] Collin C., Wade D. (1990). Assessing motor impairment after stroke: a pilot reliability study. J. Neurol. Neurosurg. Psychiatry.

[3] Lindmark B., Hamrin E. (1988). Evaluation of functional capacity after stroke as a basis for active intervention. Validation of a modified chart for motor capacity assessment. Scand. J. Rehabil. Med..

[4] Keith R.A., Granger C.V., Hamilton B.B., Sherwin F.S. (1987). The functional independence measure: a new tool for rehabilitation. Adv. Clin. Rehabil..

[5] Franchignoni F.P., Tesio L., Ricupero C., Martino M.T. (1997). Trunk control test as an early predictor of stroke rehabilitation outcome. Stroke.

[6] Cerasa A., Pignolo L., Gramigna V., Serra S., Olivadese G., Rocca F., Perrotta P., Dolce G., Quattrone A., Tonin P. (2018). Exoskeleton-robot assisted therapy in stroke patients: a lesion mapping study. Front. Neuroinf..

[7] Stinear C. (2010). Prediction of recovery of motor function after stroke. Lancet Neurol..

[8] Choo P.L., Gallagher H.L., Morris J., Pomeroy V.M., van Wijck F. (2015). Correlations between arm motor behavior and brain function following bilateral arm training after stroke: a systematic review. Brain Behav..

[9] Saleh S., Fluet G., Qiu Q., Merians A., Adamovich S.V., Tunik E. (2017). Neural patterns of reorganization after intensive robot-assisted virtual reality therapy and repetitive task practice in patients with chronic stroke. Front. Neurol..

[10] Gandolfi M., Formaggio E., Geroin C., Storti S.F., Boscolo Galazzo I., Bortolami M., Saltuari L., Picelli A., Waldner A., Manganotti P., Smania N. (2018). Quantification of upper limb motor recovery and EEG power changes after robot-assisted bilateral arm training in chronic stroke patients: a prospective pilot study. Neural Plast..

[11] Colizzi L., Lidonnici A., Pignolo L. (2009). The ARAMIS project: a concept robot and technical design. J. Rehabil. Med..

[12] Dolce G., Lucca L.F., Pignolo L. (2009). Robot-assisted rehabilitation of the paretic upper limb: rationale of the ARAMIS project. J. Rehabil. Med..

[13] Pignolo L., Lucca L.F., Basta G., Serra S., Pugliese M.E., Sannita W.G., Dolce G. (2016). A new treatment in the rehabilitation of the paretic upper limb after stroke: the ARAMIS prototype and treatment protocol. Ann. Ist. Super Sanita.

