# Supplementary material for: Data on a new neurorehabilitation approach targeting functional recovery in stroke patients
Source: Data Brief. 2019 Oct 28;27:104685. doi: 10.1016/j.dib.2019.104685 (PMC6849111; doi:10.1016/j.dib.2019.104685)
Supplement: Multimedia component 1 [file mmc1.pdf]

FUGL-MEYER ASSESSMENT UPPER EXTREMITY (FMA-UE) Assessment of sensorimotor function

ID:
Date:
Examiner:

Fugl-Meyer AR, Jaasko L, Leyman I, Olsson S, Steglind S: The post-stroke hemiplegic patient. A method for evaluation of physical performance. Scand J Rehabil Med 1975, 7:13-31.

A. UPPER EXTREMITY, sitting position

I. Reflex activity	none	can be elicited
**Flexors**: biceps and finger flexors (at least one)	0	2
**Extensors**: triceps	0	2
Subtotal I (max 4)		

II. Volitional movement within synergies, without gravitational help			none	partial	full
**Flexor synergy:** Hand from contralateral knee to ipsilateral ear. From extensor synergy (shoulder adduction/ internal rotation, elbow extension, forearm pronation) to flexor synergy (shoulder abduction/ external rotation, elbow flexion, forearm supination).	Shoulder	retraction	0	1	2
		elevation	0	1	2
		abduction (90°)	0	1	2
		external rotation	0	1	2
	Elbow	flexion	0	1	2
	Forearm	supination	0	1	2
**Extensor synergy:** Hand from ipsilateral ear to the contralateral knee	Shoulder	adduction/internal rotation	0	1	2
	Elbow	extension	0	1	2
	Forearm	pronation	0	1	2
		Subtotal II (max 18)			

III. Volitional movement mixing synergies, without compensation		none	partial	full
**Hand to lumbar spine**<br>hand on lap	cannot perform or hand in front of ant-sup iliac spine	0		
	hand behind ant-sup iliac spine (without compensation)		1	
	hand to lumbar spine (without compensation)			2
**Shoulder flexion 0°- 90°**<br>elbow at 0°<br>pronation-supination 0°	immediate abduction or elbow flexion	0		
	abduction or elbow flexion during movement		1	
	flexion 90°, no shoulder abduction or elbow flexion			2
**Pronation-supination**<br>elbow at 90°<br>shoulder at 0°	no pronation/supination, starting position impossible	0		
	limited pronation/supination, maintains starting position		1	
	full pronation/supination, maintains starting position			2
	Subtotal III (max 6)			

IV. Volitional movement with little or no synergy		none	partial	full
**Shoulder abduction 0 - 90°**<br>elbow at 0°<br>forearm neutral	immediate supination or elbow flexion	0		
	supination or elbow flexion during movement		1	
	abduction 90°, maintains extension and pronation			2
**Shoulder flexion 90° - 180°**<br>elbow at 0°<br>pronation-supination 0°	immediate abduction or elbow flexion	0		
	abduction or elbow flexion during movement		1	
	flexion 180°, no shoulder abduction or elbow flexion			2
**Pronation/supination**<br>elbow at 0°<br>shoulder at 30°- 90° flexion	no pronation/supination, starting position impossible	0		
	limited pronation/supination, maintains start position		1	
	full pronation/supination, maintains starting position			2
	Subtotal IV (max 6)			

V. Normal reflex activity assessed only if full score of 6 points is achieved in part IV; compare with the unaffected side		hyper	lively	normal
Biceps, triceps, finger flexors	2 of 3 reflexes markedly hyperactive	0		
	1 reflex markedly hyperactive or at least 2 reflexes lively		1	
	maximum of 1 reflex lively, none hyperactive			2
	Subtotal V (max 2)			
	**Total A** (max 36)			

GÖTEBORGS UNIVERSITET

**B. WRIST** support may be provided at the elbow to take or hold the starting position, no support at wrist, check the passive range of motion prior testing		none	partial	full
**Stability at 15° dorsiflexion**<br>elbow at 90°, forearm pronated<br>shoulder at 0°	less than 15° active dorsiflexion<br>dorsiflexion 15°, no resistance tolerated<br>maintains dorsiflexion against resistance	0	1	2
**Repeated dorsifexion / volar flexion**<br>elbow at 90°, forearm pronated<br>shoulder at 0°, slight finger flexion	cannot perform volitionally<br>limited active range of motion<br>full active range of motion, smoothly	0	1	2
**Stability at 15° dorsiflexion**<br>elbow at 0°, forearm pronated<br>slight shoulder flexion/abduction	less than 15° active dorsiflexion<br>dorsiflexion 15°, no resistance tolerated<br>maintains dorsiflexion against resistance	0	1	2
**Repeated dorsifexion / volar flexion**<br>elbow at 0°, forearm pronated<br>slight shoulder flexion/abduction	cannot perform volitionally<br>limited active range of motion<br>full active range of motion, smoothly	0	1	2
**Circumduction**<br>elbow at 90°, forearm pronated<br>shoulder at 0°	cannot perform volitionally<br>jerky movement or incomplete<br>complete and smooth circumduction	0	1	2
	**Total B** (max 10)			

**C. HAND** support may be provided at the elbow to keep 90° flexion, no support at the wrist, compare with unaffected hand, the objects are interposed, active grasp		none	partial	full
**Mass flexion**<br>from full active or passive extension		0	1	2
**Mass extension**<br>from full active or passive flexion		0	1	2
**GRASP**				
**a. Hook grasp**<br>flexion in PIP and DIP (digits II-V),<br>extension in MCP II-V	cannot be performed<br>can hold position but weak<br>maintains position against resistance	0	1	2
**b. Thumb adduction**<br>1-st CMC, MCP, IP at 0°, scrap of paper<br>between thumb and 2-nd MCP joint	cannot be performed<br>can hold paper but not against tug<br>can hold paper against a tug	0	1	2
**c. Pincer grasp, opposition**<br>pulpa of the thumb against the pulpa of<br>2-nd finger, pencil, tug upward	cannot be performed<br>can hold pencil but not against tug<br>can hold pencil against a tug	0	1	2
**d. Cylinder grasp**<br>cylinder shaped object (small can)<br>tug upward, opposition of thumb and<br>fingers	cannot be performed<br>can hold cylinder but not against tug<br>can hold cylinder against a tug	0	1	2
**e. Spherical grasp**<br>fingers in abduction/flexion, thumb<br>opposed, tennis ball, tug away	cannot be performed<br>can hold ball but not against tug<br>can hold ball against a tug	0	1	2
	**Total C** (max 14)			

GÖTEBORGS UNIVERSITET

**D. COORDINATION/SPEED**, sitting, after one trial with both arms, eyes closed, tip of the index finger from knee to nose, 5 times as fast as possible		marked	slight	none
**Tremor**	at least 1 completed movement	0	1	2
**Dysmetria**	pronounced or unsystematic<br>slight and systematic<br>no dysmetria	0	1	2
		**≥ 6s**	**2 - 5s**	**< 2s**
**Time**<br>start and end with the<br>hand on the knee	6 or more seconds slower than unaffected side<br>2-5 seconds slower than unaffected side<br>less than 2 seconds difference	0	1	2
	**Total D** (max 6)			

**TOTAL A-D** (max 66)	

**H. SENSATION**, upper extremity<br>eyes closed, compared with the unaffected side		**anesthesia**	**hypoesthesia or dysesthesia**	**normal**
**Light touch**	upper arm, forearm<br>palmary surface of the hand	0<br>0	1<br>1	2<br>2
		**less than 3/4 correct or absence**	**3/4 correct or considerable difference**	**correct 100%, little or no difference**
**Position**<br>small alterations in the position	shoulder<br>elbow<br>wrist<br>thumb (IP-joint)	0<br>0<br>0<br>0	1<br>1<br>1<br>1	2<br>2<br>2<br>2
			**Total H** (max12)	

**I. PASSIVE JOINT MOTION**, upper extremity,<br>sitting position, compare with the unaffected side				**J. JOINT PAIN** during passive motion, upper extremity		
	only few degrees (less than 10° in shoulder)	decreased	normal	pronounced pain during movement or very marked pain at the end of the movement	some pain	no pain
**Shoulder**						
Flexion (0° - 180°)	0	1	2	0	1	2
Abduction (0°-90°)	0	1	2	0	1	2
External rotation	0	1	2	0	1	2
Internal rotation	0	1	2	0	1	2
**Elbow**						
Flexion	0	1	2	0	1	2
Extension	0	1	2	0	1	2
**Forearm**						
Pronation	0	1	2	0	1	2
Supination	0	1	2	0	1	2
**Wrist**						
Flexion	0	1	2	0	1	2
Extension	0	1	2	0	1	2
**Fingers**						
Flexion	0	1	2	0	1	2
Extension	0	1	2	0	1	2
**Total** (max 24)				**Total** (max 24)		

GÖTEBORGS UNIVERSITET

**A. UPPER EXTREMITY**	/36
**B. WRIST**	/10
**C. HAND**	/14
**D. COORDINATION / SPEED**	/ 6
**TOTAL A-D (motor function)**	/66

**H. SENSATION**	/12
**I. PASSIVE JOINT MOTION**	/24
**J. JOINT PAIN**	/24
