# Supplementary material for: Data on a new neurorehabilitation approach targeting functional recovery in stroke patients
Source: Data Brief. 2019 Oct 28;27:104685. doi: 10.1016/j.dib.2019.104685 (PMC6849111; doi:10.1016/j.dib.2019.104685)
Supplement: Multimedia component 2 [file mmc2.pdf]

MOTRICITY INDEX

AND

TRUNK CONTROL TEST

PATIENT'S NAME:

HOSPITAL NUMBER:

Date										
Side tested										
**ARM** TO BE CONDUCTED IN SITTING POSITION<br>1. Pinch grip<br>*2.5cm cube between thumb and forefinger.*										
2. Elbow flexion<br>*from 90°, voluntary contraction/movement.*										
3. Shoulder abduction<br>*from against chest*										
**LEG** TO BE CONDUCTED IN SITTING POSITION<br>4. Ankle dorsiflexion<br>*from plantar flexed position.*										
5. Knee extension<br>*from 90°, voluntary contraction/movement.*										
6. Hip flexion<br>*usually from 90.°*										
**ARM SCORE** (1+2+3)										
**LEG SCORE** (4+5+6)										
**SIDE SCORE** (Arm + leg)/2										
**TRUNK CONTROL TEST** ON THE BED<br>7. Rolling to weak side										
8. Rolling to strong side										
9. Sitting up from lying down										
10. Balance in sitting position<br>*On side of bed.*										
**TRUNK SCORE** (7+8+9+10)										

TEST 1 (Pinch grip)
- *0 = No movement*
- *11 = Beginnings of prehension*
- *19 = Grips cube but unable to hold against gravity.*
- *22 = Grips cube, held against gravity but not against weak pull.*
- *26 = Grips cube against pull but weaker than other/normal side.*
- *33 = Normal pinch grip.*

TESTS 2 - 6
- *0 = No movement*
- *9 = Palpable contraction in muscle but no movement.*
- *14 = Movement seen but not full range/not against gravity.*
- *19 = Full range against gravity, not against resistance.*
- *25 = Movement against resistance but weaker than other side.*
- *33 = Normal power*

TRUNK CONTROL TEST
- *0 = Unable to do on own.*
- *12 = Able to do but only with non-muscular help (pulling on bedclothes,using arms to steady self when sitting, pulling up on monkey pole etc).*
- *25 = Normal*
