# Supplementary material for: Data on a new neurorehabilitation approach targeting functional recovery in stroke patients
Source: Data Brief. 2019 Oct 28;27:104685. doi: 10.1016/j.dib.2019.104685 (PMC6849111; doi:10.1016/j.dib.2019.104685)
Supplement: Multimedia component 4 [file mmc4.pdf]

FIM™ Instrument

LEVELS		
	7 Complete Independence (timely, safely)<br>6 Modified Independence (device)	NO HELPER
	**Modified Dependence**<br>5 Supervision (subject = 100%)<br>4 Minimal Assistance (subject = 75%+)<br>3 Moderate Assistance (subject = 50%+)	HELPER
	**Complete Dependence**<br>2 Maximal Assistance (subject = 25%)<br>1 Total Assistance (subject = less than 25%)	

	ADMISSION	DISCHARGE	FOLLOW-UP
**Self-Care**			
A. Eating			
B. Grooming			
C. Bathing			
D. Dressing - Upper Body			
E. Dressing - Lower Body			
F. Toileting			
**Sphincter Control**			
G. Bladder Management			
H. Bowel Management			
**Transfers**			
I. Bed, Chair, Wheelchair			
J. Toilet			
K. Tub, Shower			
**Locomotion**			
L. Walk/Wheelchair	W Walk<br>C Wheelchair<br>B Both	W Walk<br>C Wheelchair<br>B Both	W Walk<br>C Wheelchair<br>B Both
M. Stairs			
Motor Subtotal Rating			
**Communication**			
N. Comprehension	A Auditory<br>V Visual<br>B Both	A Auditory<br>V Visual<br>B Both	A Auditory<br>V Visual<br>B Both
O. Expression	V Vocal<br>N Nonvocal<br>B Both	V Vocal<br>N Nonvocal<br>B Both	V Vocal<br>N Nonvocal<br>B Both
**Social Cognition**			
P. Social Interaction			
Q. Problem Solving			
R. Memory			
Cognitive Subtotal Score			
Total FIM ™ Score			

Note: Leave no blanks. Enter 1 if patient is not testable due to risk.

FIM™ Instrument. Copyright© 1997 Uniform Data System for Medical Rehabilitation, a division of UB Foundation Activities, Inc.
Reprinted with permission of UDSMR, University at Buffalo, 232 Parker Hall, 3435 Main Street, Buffalo, NY 14214.
