# Supplementary material for: Data on a new neurorehabilitation approach targeting functional recovery in stroke patients
Source: Data Brief. 2019 Oct 28;27:104685. doi: 10.1016/j.dib.2019.104685 (PMC6849111; doi:10.1016/j.dib.2019.104685)

**SUPPLEMENTARY MATERIALS**

1. **Data on a new neurorehabilitation approach targeting functional recovery in stroke patients.**
2. Loris Pignolo^1^, Sebastiano Serra^1^, Giuseppina Basta^1^, Simone Carozzo^1^, Francesco Arcuri^1^, Marialuigina Pignataro^1^, Irene Ciancarelli^2^, Paolo Tonin^2^. Antonio Cerasa^1,3*^;

SURVEY/QUESTIONNARIE

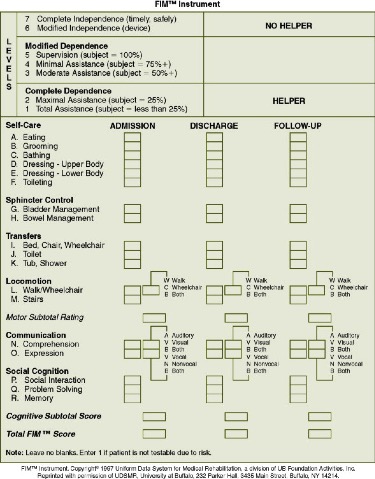

Supplement: Multimedia component 5 [file mmc5.docx]
